# AAV‐mediated expression of secreted and transmembrane αKlotho isoforms rescues relevant aging hallmarks in senescent SAMP8 mice

**DOI:** 10.1111/acel.13581

**Published:** 2022-03-10

**Authors:** J. Roig‐Soriano, C. Griñán‐Ferré, J. F. Espinosa‐Parrilla, C. R. Abraham, A. Bosch, M. Pallàs, Miguel Chillón

**Affiliations:** ^1^ Institut de Neurociènces (INc) Department of Biochemistry and Molecular Biology Universitat Autònoma Barcelona Bellaterra Spain; ^2^ Pharmacology Section Department of Pharmacology, Toxicology, and Therapeutic Chemistry Faculty of Pharmacy and Food Sciences Institut de Neurosciències‐Universitat de Barcelona (NeuroUB) Barcelona Spain; ^3^ Department of Pharmacology and Experimental Therapeutics Boston University School of Medicine Boston Massachusetts USA; ^4^ Vall d'Hebron Institut de Recerca (VHIR) Barcelona Spain; ^5^ Unitat producció de Vectors (UPV) Universitat Autònoma Barcelona Bellaterra Spain; ^6^ Centro de Investigación Biomédica en Red sobre Enfermedades Neurodegenerativas (CIBERNED) Instituto de Salud Carlos III Madrid Spain; ^7^ Institució Catalana de Recerca i Estudis Avançats (ICREA) Passeig Lluis Companys Barcelona Spain

**Keywords:** AAV9, anti‐aging, epigenetics, Klotho, neurodegeneration, osteoporosis, SAMP8, senescence

## Abstract

Senescence represents a stage in life associated with elevated incidence of morbidity and increased risk of mortality due to the accumulation of molecular alterations and tissue dysfunction, promoting a decrease in the organism's protective systems. Thus, aging presents molecular and biological hallmarks, which include chronic inflammation, epigenetic alterations, neuronal dysfunction, and worsening of physical status. In this context, we explored the AAV9‐mediated expression of the two main isoforms of the aging‐protective factor Klotho (KL) as a strategy to prevent these general age‐related features using the senescence‐accelerated mouse prone 8 (SAMP8) model. Both secreted and transmembrane KL isoforms improved cognitive performance, physical state parameters, and different molecular variables associated with aging. Epigenetic landscape was recovered for the analyzed global markers DNA methylation (5‐mC), hydroxymethylation (5‐hmC), and restoration occurred in the acetylation levels of H3 and H4. Gene expression of pro‐ and anti‐inflammatory mediators in central nervous system such as TNF‐α and IL‐10, respectively, had improved levels, which were comparable to the senescence‐accelerated‐mouse resistant 1 (SAMR1) healthy control. Additionally, this improvement in neuroinflammation was supported by changes in the histological markers Iba1, GFAP, and SA β‐gal. Furthermore, bone tissue structural variables, especially altered during senescence, recovered in SAMP8 mice to SAMR1 control values after treatment with both KL isoforms. This work presents evidence of the beneficial pleiotropic role of Klotho as an anti‐aging therapy as well as new specific functions of the KL isoforms for the epigenetic regulation and aged bone structure alteration in an aging mouse model.

## Graphical abstract

Intraventricular administration of AAV vectors expressing secreted and transmembrane Klotho isoforms, rescued accelerated aging phenotype of SAMP8 mice. An improvement in cognitive and physical performance, recovery of epigenetic, inflammatory and senescence markers, as well as structural changes in long bones of these mice was detected.

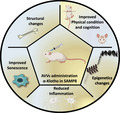

## 1 INTRODUCTION

Due to the increasing proportion of the aged population in modern societies, research in senescence hallmarks, and pleiotropic methods to reverse them are a very active field of research (Lopez‐Otin et al., 2013). Senescence is understood as a gradual degenerative process that after adulthood leads to tissue dysfunction due to the deregulation of critical cellular events such as epigenetic mechanisms, mitochondrial function, oxidative stress (OS), inflammation, intercellular communication, among others (Delgado‐Morales et al., 2017; McHugh et al., 2018). Those alterations are due to both genetic and environmental factors, which generate long‐term accumulative damage and increase vulnerability to death (Delgado‐Morales et al., 2017; Lopez‐Otin et al., 2013).

One of the most critically affected systems is the epigenetic‐mediated gene regulation, a well‐known hallmark of aging that leads to genomic instability and gene expression deregulation (Delgado‐Morales et al., 2017). Epigenetic modulation includes diverse molecular processes, including DNA methylation (5‐mC), hydroxymethylation (5‐hmC), histone modifications, and non‐coding RNA (Grinan‐Ferre et al., 2018). It is well established that variations in global DNA methylation (5‐mC) and hydroxymethylation (5‐hmC) epigenetic patterns can influence gene expression and alter neuronal function, promoting the progression of age‐related chronic and neurodegenerative diseases in mouse models and humans (Coppieters et al., 2014; Grinan‐Ferre et al., 2018). Similarly, the inflammatory response is also exacerbated during aging, resulting in chronic damage in the central nervous system (CNS) (Kinney et al., 2018). Cellular alterations such as microgliosis and astrocytosis, as well as dysregulation of inflammatory markers such as tumor necrosis factor‐alpha (TNF‐α), interleukin‐1beta (IL‐1β), or interleukin‐6 (IL‐6), among others, have also been described during normal aging (Zoller et al., 2018). These changes, together with the previously mentioned deregulations, directly affect cognition and memory formation (Grinan‐Ferre et al., 2016, 2018).

Tissue degeneration is another feature associated with aging. Skeletal tissue is especially altered during this period, characterized by a progressive loss in bone mineral density (BMD) and alterations in the shape and structure of bone components (Ferguson et al., 2003; Riggs et al., 2004). All these changes result in reduced bone quality, including a reduction in bone strength and flexibility, leading to an increased prevalence of fractures (Ammann & Rizzoli, 2003; Silva et al., 2002).

Besides stress factors that could enhance senescence, some molecules with anti‐aging properties can decelerate or even rescue previously mentioned aging hallmarks. Among them, the chronokine Klotho (KL) is an exciting candidate due to its pleiotropic anti‐aging protection (Abraham et al., 2016; Dubal et al., 2015; Kuro‐o et al., 1997). KL is involved in pathways that drive age‐related chronic disorders such as kidney disease, tissue dysfunction, neurodegenerative diseases, and cancer, among others (Moos et al., 2020; Semba et al., 2014). The Klotho gene is predominantly expressed in the kidney and choroid plexus in the brain as two main transcripts (Shiraki‐Iida et al., 1998). Full‐length mRNA translates into a single‐pass transmembrane protein (m‐KL), composed of two extracellular domains (KL1 and KL2). The extracellular domains can be released from the membrane by protease‐mediated shedding, generating soluble, circulating processed Klotho (p‐KL) (Chen et al., 2014). The alternatively spliced mRNA possesses a premature STOP codon and generates a secreted protein (s‐KL) containing just the KL1 domain and an additional 15 amino acids at its C‐terminus (Shiraki‐Iida et al., 1998). Specific functions of s‐KL isoform have not been extensively studied. However, our previous studies indicate that s‐KL is active and possesses potent anti‐aging properties in the CNS (Masso et al., 2018).

Among other functions, KL isoforms are key regulators of Ca^+2^ and PO_4_^−3^ ion mineral homeostasis (Chen et al., 2018) and generate protection against oxidative and inflammatory stress (Yamamoto et al., 2005), both pathways being involved in health and senescence protection (Lopez‐Otin et al., 2013). Interestingly, several studies have demonstrated that high levels of KL are associated with enhanced cognition (Dubal et al., 2015; Masso et al., 2018), health status, and longevity in both mice and humans (Arking et al., 2002; Kurosu et al., 2005). However, the KL promoter is susceptible to age‐dependent methylation, reducing its expression during aging progression (King et al., 2012). This hypermethylation of the promoter makes the recovery of KL expression a thought‐provoking strategy for addressing age‐associated conditions.

The senescence‐accelerated prone mouse 8 (SAMP8) and its healthy control, the senescence‐accelerated mouse resistant 1 (SAMR1), are well‐established murine models for the study of age‐related cognitive decline (Akiguchi et al., 2017). SAMP8 mice start developing age‐related deficits since they are 2‐month‐old, becoming clearly different from SAMR1 animals at the age of 5 months. Features seen during SAMP8 mice aging resemble human senescence progression, including the appearance of memory and cognitive deficits (Grinan‐Ferre et al., 2018), elevated reactive oxidative species (ROS) levels (Zhou et al., 2018), altered gene expression due to epigenetic control deregulation (Grinan‐Ferre et al., 2016), osteoporosis (Chen et al., 2009), and histological features associated with neuronal damage and inflammation (Akiguchi et al., 2017).

Specifically, the present study assessed the physical, behavior, and cognitive conditions after the individual expression of the two main KL transcripts in SAMP8 mice to rise exogenously KL’s protective properties in this mouse model. To this end, we analyzed age‐related features, including cognitive and physical decline, epigenetic markers, histological state, and long bone integrity. Furthermore, to reveal isoform‐specific properties in aged mice, we separately expressed the two main KL transcripts allowing for the first time a comparative analysis between them.

## 2 EXPERIMENTAL PROCEDURES

### 2.1 Animal housing

SAMR1 (*n* = 9) and SAMP8 (*n* = 29) male mice (7‐month‐old) were used to perform behavioral and molecular analyses. The animals were divided randomly into four groups: SAMR1 Control (SR1 Null) (*n* = 9), SAMP8 Control (SP8 Null) (*n* = 9), both injected with AAV9 Null, SAMP8 injected with AAV9 s‐KL (SP8 s‐KL) (*n* = 11), and SAMP8 injected with AAV9 m‐KL (SP8 m‐KL) (*n* = 9). Klotho constructs (s‐KL and m‐KL) derived from the Klotho mouse gene. Mice had free access to food and water and were kept under standard temperature conditions (22±2°C) and a 12‐h light/dark cycle (300 lux/0 lux). We worked with 7‐month‐old mice because compared with SAMR1 mice, SAMP8 mice show clear age‐related deficits resembling human senescence progression. Animals were deeply anesthetized by intraperitoneal injection of 10 mg/kg of ketamine (Imalgene 500, Rhone‐Merieux) and 1 mg/kg of xylazine (Rompun, Bayer) diluted in NaCl 0.9%. Intracerebroventricular stereotaxic injections of AAV vectors were performed in the right hemisphere at coordinates, −0.2 mm Antero‐posterior, −2 mm Dorso‐ventral, and +1 mm medio‐lateral from bregma. The vector dose was 1 × 10^11^ viral genomes per animal in 6 μl, administered at a 0.5 μl/min speed using an ultramicropump (WorldPrecision Instruments).

Adeno‐associated viruses (AAV) serotype 9 were generated in HEK293 cells by the triple transfection method at the Unitat de Producció de Vectors (UPV) at Universitat Autònoma de Barcelona following a protocol described previously (Piedra et al., 2015).

All experimental procedures involving animals were performed following standard ethical guidelines of the European Communities Council Directive 86/609/EEC and by the Institutional Animal Care and Use Committee of the University of Barcelona (670/14/8102, approved at 11/14/2014) and by Generalitat de Catalunya (10291, approved 1/28/2018).

### 2.2 Behavioral tests

Before the behavioral tests procedure, brief examination was done to ensure animals did not presented vision or locomotor problems. All animals went through the following tests allowing one day of recovery between tests.

### 2.3 Horizontal wooden bar

A circular wooden bar with 1 cm of diameter was horizontally placed 40 cm above a soft floor made of expanded polystyrene. Mice were carefully suspended on the bar by their upper limbs, a maximum test length of 40 s. Time spent on the bar (resistance) and distance walked along the bar (coordination) were recorded. Two trials per animal were done, and the best performance was selected.

### 2.4 Open field test (OFT)

The OFT was performed as previously described (Archer, 1973). The floor was divided into two areas defined as the center and peripheral zone. Behavior was scored with SMART^®^ ver.3.0 software (Panlab), and each trial was recorded for later analysis using a camera situated above the apparatus. Mice were placed at the center and allowed to explore the white polywood box (50 × 50 × 25 cm) for 5 min. Afterward, the mice were returned to their home cages, and the OFT apparatus was cleaned with 70% ethanol (EtOH). The parameters scored included center staying duration, rearings, defecations, urinations, and the distance travelled, calculated as the sum of global distance travelled in the open field arena for 5 min.

### 2.5 Object location test (OLT)

The object location test (OLT) is a well‐established task based on the spontaneous tendency of rodents to spend more time exploring a novel object location than a familiar object location, as well as to recognize when an object has been relocated. The test was carried out for 3 days in a wooden box (50 × 50 × 25 cm), in which three walls were white except one that was black. The first day, the box was empty, and the animals just habituated to the open field arena for 10 min. The second day, two objects were placed in front of the black wall, equidistant from each other and the wall. The objects were 10‐cm high and identical. The animals were placed into the open field arena and allowed to explore both objects and surroundings, for 10 min. Afterward, animals were returned to their home cages, and the OLT apparatus was cleaned with 70% ethanol. On the third day, one object was moved in front of the white wall to test the spatial memory. Trials were recorded using a camera mounted above the open field area, and the total exploration time was determined by scoring the amount of time (seconds) spent sniffing the object in the new location (TN) and the object in the old location (TO). To evaluate the cognitive performance, the discrimination index (DI) was calculated, which is defined as (TN − TO)/(TN + TO).

### 2.6 Novel object recognition test (NORT)

In brief, mice were placed in a 90°, two‐arm, (25 × 20 × 5 cm) black maze. The walls could be removed for easy cleaning. Light intensity in mid‐field was 30 lux. Before the memory test, a 3‐day‐habituation was performed in which the mice were placed individually in the apparatus for 10 min. On Day 4, the familiarization phase took place, in which the animals were placed in the maze in the presence of two identical, novel objects (A + A) or (B + B) located at the end of each maze. During this 10‐min acquisition trial, the mice were allowed to explore the two identical objects. After 2 and 24 h from that trial, the animals were submitted to a 10‐min retention trial in which one of the two objects was replaced by a novel one. The behavior of the animals was recorded during the 2 and 24 h retention trials using a camera fixed to the ceiling and situated above the apparatus. The amount of object exploration was defined as sniffing or touching the objects with nose and/or forepaws. The time spent exploring the new object (TN) and the time spent exploring the old one (TO) were measured manually, and the discrimination index (DI) was calculated as (TN − TO)/(TN + TO) (Ennaceur & Delacour, 1988). For the elimination of olfactory cues, 70% ethanol was used to clean the removable arms and objects after each trial.

### 2.7 Elevated plus maze (EPM)

The elevated plus maze (EPM) consisted of two open arms (30 × 5 × 15 cm); two enclosed arms (30 × 5 × 15 cm) positioned 40 cm above the floor. The junction of four arms formed a central square platform (5 × 5 cm). Each animal was placed on the central platform facing one of the open arms and was allowed to explore freely for 5 min. The behavior parameters evaluated were the number of entries in the open arms and the percentage of time spent in the open and closed arms, time spent in the center zone, rearings, defecations, urination, among others, scored with SMART^®^ vers.3.0 software (Panlab).

### 2.8 Brain processing

Animals were euthanized by cervical dislocation 3 days after the last behavioral test. Brains were immediately removed from the skull. The hippocampus and cortex were then isolated and frozen in powdered dry ice. They were maintained at −80°C until use for gene expression and epigenetic changes analysis described below.

### 2.9 RNA extraction and gene expression analysis

Total RNA isolation was carried out using TRIsure^TM^ reagent following the manufacturer's instructions (Bioline Reagent). The liver, hippocampus, or cortex samples were homogenized using TissueLyser LT sample disruption apparatus (QIAGEN). RNA quantity and purity were measured with NanoDrop™ 1000 Spectrophotometer (Thermo Scientific). RNA retrotranscription was done using iScript™ Advanced cDNA Synthesis Kit (Bio‐rad). Gene expression was analyzed by real‐time quantitative PCR (RT‐qPCR) on a Bio‐Rad CFX‐384 PCR machine at the Analysis and Photodocumentation Service of the Universitat Autonòma de Barcelona. Each reaction contained 25 ng of cDNA, 7.5 μl of iTaqTM Universal SYBR Green Supermix (Bio‐Rad), and a primer concentration of 0.2 nM, with a final reaction volume of 15 μl. Primers used are listed in Table S2.

The analysis of qPCR data was done following the ΔΔCt method. Cycle threshold (Ct) were normalized subtracting from each experimental Ct, the difference of its housekeeping (HK) Ct in regard to the HK’s average value. HK genes used were β‐actin and GAPDH. Melting curves were also analyzed to ensure unique amplificon generation. Each sample (*n* = 6 per group) was tested at least in duplicates, and Cts higher than 37 cycles were considered as not amplified.

### 2.10 Global epigenetic changes analysis

Isolation of genomic DNA from the hippocampus tissue (*n* = 3 per group) was conducted using the FitAmp^TM^ Blood and Cultured Cell DNA Extraction Kit according to the manufacturer's instructions. Then, Methylflash Methylated DNA Quantification Kit (Epigentek) and MethylFlash HydroxyMethylated DNA Quantification Kit were used in order to detect epigenetic markers. Briefly, these kits are based on specific antibody detection of 5‐mC and 5‐hmC residues, which trigger an ELISA‐like reaction that allows colorimetric quantification by reading absorbance at 450 nm using a Microplate Photometer. The absolute amount of methylated or hydroxymethylated DNA (proportional to the Optical Density [OD] intensity) was measured and quantified using a standard curve plotting OD values vs. five serial dilutions of a control methylated and hydroxymethylated DNA (0.5–10 ng).

### 2.11 Global histone acetylation H3 and H4 quantification

Histone extracts from the hippocampus tissue (*n* = 3 per group) were prepared by using a total histone extraction kit (Epigentek) according to the manufacturer's protocol. Detection of global histone H3/H4 acetylation levels was measured using the EpiQuik™ global histone H3/H4 acetylation assay kit (Epigentek). Briefly, histone proteins (1–2 μg) were added to the strip wells. Acetylated histone H3/H4 was detected with a high‐affinity antibody, and the ratios and amounts of acetylated histone H3/H4 were displayed with a horseradish peroxidase‐conjugated secondary antibody color development system. The color was measured by reading absorbance at 450 nm using a Microplate Photometer.

### 2.12 Histology

Animals were perfused by intracardiac injection with cold PBS solution followed with a solution of PFA 4%. After fixation, brains were dissected and submerged in PFA 4% for 24 h. Samples were washed using PBS and then incubated in 30% sucrose at 4°C for cryoprotection. Brains were embedded in OCT (Tissue‐Tek) and frozen on dry ice. Slices of 10 μm thickness were obtained with a cryostat Leica CM3050S (Leica).

Staining procedures consisted of permeabilization with 0.3% Tris‐Tween buffer solution for 20 min or with methanol (Panreac) for 10 s, followed by 1h blocking with 0.5% bovine serum albumin (BSA) in TBST. Primary antibody incubation was done at 4°C overnight (ON). Next, three rounds of 10 min long wash with TBST at room temperature (RT) were done, followed by 1h incubation at RT with the secondary antibody. Samples were subsequently washed with other three rounds of 10 min and a 5 min long incubation with 1:500 Hoescht dilution to stain nuclei. Finally, slides were washed and mounted with Fluoromount (Thermo Scientific). A list of antibodies used is shown in Table S3.

Images were obtained with an optical epifluorescence microscope Nikon Eclipse 90i or confocal laser scanning microscope (Zeiss LSM 700). Pictures were analyzed using ImageJ software to obtain optical density (OD), corrected total cell fluorescence (CTCF), cell number, and mean cell size values.

### 2.13 Bone study

Right legs of perfused animals were isolated and placed in a PFA 4% solution for preservation. Tibias were dissected, muscle tissue was removed, and bones placed in PBS solution with 0.05% sodium azide (NaN_3_). MicroCT analysis was done with a SkyScan 1272 (Bruker) computerized microtomography imaging system at the Centre de Recerca en Ciència i Enginyeria Multiescala de Barcelona (CRCEMB) at Universitat Politècnica de Catalunya (UPC).

Images were reconstructed with the NRecon v1.6 (Bruker) program and analyzed with the CT‐Analyser v1.13 image program (Bruker). Cortical bone analysis was done with 100 slices by manually selecting volumes of interest (VOIs), with a binary threshold of 50–255. Analyzed image intervals started from peronei insertion toward upper tibial epiphysis. For trabecular bone analysis, 150 slices were analyzed with a binary threshold of 20–255. All selected interval of images started 100 VOIs from the femoral distal growth plate toward tibial diaphysis. Finally, 3D representations of the bones were obtained with the CTVox v3.3 program (Bruker).

Mineral density was calculated with the CT‐Analyzer v1.13 program calibrating bone absorbance with two 2mm diameter hydroxyapatite phantoms (Bruker‐MicroCT) of known density of 0.25 and 0.75 g/cm^3^.

### 2.14 Statistical analysis

Statistical analysis and graphic representation were done with GraphPad Prism ver.6 (GraphPad Software). Statistical differences between groups were analyzed with a two‐tailed unpaired Student's *t*‐test when comparing two groups, and one‐way analysis of variance (ANOVA), followed by Tukey as a post‐hoc analysis when it was necessary. Data are expressed as mean ± standard error of the mean (SEM). Statistical difference was accepted when *p* values were ≤0.05, and outliers were detected by Grubb's test and removed from the analysis.

## 3 RESULTS

### 3.1 AAV administration efficiently expressed KL in SAMP8 animals

**FIGURE 1:**
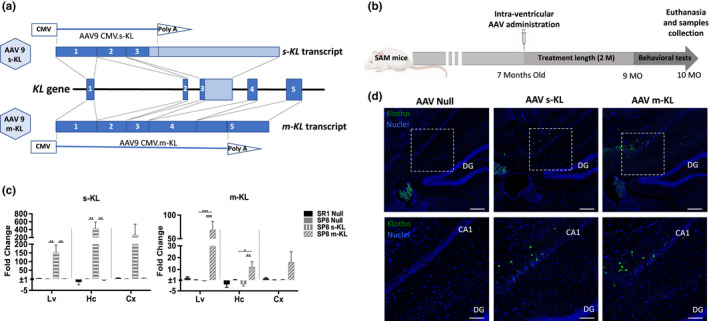
(a) αKL gene structure, transcription and AAV9 constructs. (b) Experimental design. (c) qPCR analysis of KL isoforms expression levels in the liver (Lv), hippocampus (Hc), and cortex (Cx) of animals treated with AAV9 Null, s‐KL or m‐KL. Data represented as fold change compared to the SP8 Null group. Mean ± standard error of the mean (SEM), *n* = 6; *p < 0.05; **p < 0.01; ***p < 0.001. (d) Histological analysis of treated brains stained with KL (1:100) (green) and nuclei stained with Hoechst (1:500) (blue). Scale bar for immunohistochemical images is 100 μm (first row) and 50 μm (second row)

Three different adeno‐associated virus (AAV) vectors were generated containing the sequence of the full‐length transmembrane Klotho, the secreted isoform and a Null control vector. AAV9 serotype in known to extravasate to bloodstream after intra‐CNS injection (Schuster et al., 2014); so, it was the serotype selected for its capacity to efficiently transduce neurons but also liver cells (Figure 1a). Intracerebroventricular administration was done in 7‐month‐old mice, and a therapeutic period of 10 weeks was set, finishing the experiment when animals were 10‐month‐old (Figure 1b). The effectiveness of AAV9‐mediated expression of m‐KL and s‐KL was evaluated by RNA analysis from different tissues. As seen in Figure 1c, both AAV9 s‐KL and m‐KL mediated strong αKlotho expression two months after AAV injection in the different areas analyzed. Vector expression was associated with an increase in KL protein in the hippocampu*s* (Figure 1d), area of particular interest due to its importance in learning and memory formation.

### 3.2 KL expression improved physical condition and cognitive performance without changes in age‐related behaviors in treated SAMP8 mice

**FIGURE 2:**
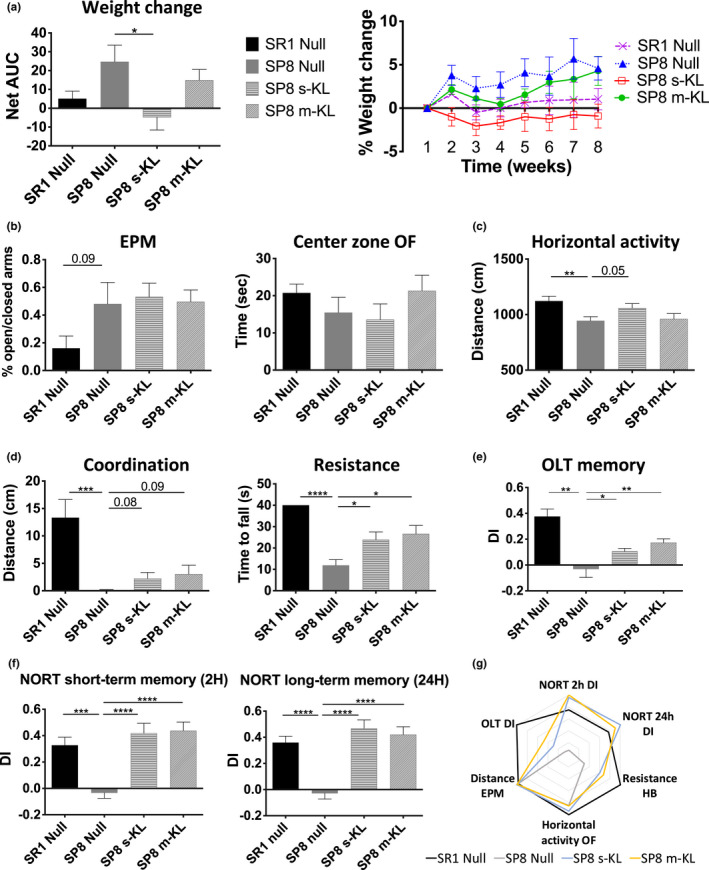
(a) Body weight represented as the % weight change per week. Statistical analysis was done with the area under the curve value. (b) Percentage of the time spent in open/closed arms in the elevated plus maze test (EPM). Time spent in the central zone of OF presented as seconds (s). (c) Mean horizontal activity during OF test. (d) Coordination and strength of treated mice measured with the horizontal wooden bar test. (e) Moved object discrimination index measured in OLT. (f) Discrimination index of the new object in NORT. The first graph represents short‐term memory and second long‐term memory establishment. (g) Polygonal graph presenting a summary of relevant behavioral parameters. Mean ± standard error of the mean (SEM), *n* = 9–11; &*p* = 0.05 *p < 0.05; **p < 0.01, ***p < 0.001, ****p < 0.0001

The effect of AAV‐mediated KL isoforms expression on the physical and cognitive state was evaluated by analyzing body weight, behavioral indicators, and activity parameters. Briefly, a 5% body weight increase was detected in SAMP8 animals during the experiment length. In contrast, both control SAMR1 (SR1) and s‐KL treated animals maintained the average body weight constant (Figure 2a). No other significant differences in anxiety indicators were observed in the elevated plus maze (EPM) or the open field test (OFT) (Figure 2b) (Tables 1 and 2). Physical tests indicated a significant increase in horizontal activity of SAMP8 s‐KL treated group compared with SAMP8 Null in the OFT (Figure 2c), as well as a significant increase in resistance and partial recovery in coordination in horizontal wooden bar test after KL treatments (Figure 2d). After two months of treatment, memory was also analyzed using object location test (OLT) and novel object recognition test (NORT). A partial but significant improvement in OLT performance after KL treatment was observed (Figure 2e). Notably, both s‐KL and m‐KL treatments increased mice capacity to recognize the new object during NORT. These differences were statistically significant when compared with the obtained discrimination index (Figure 2f). Finally, differences in physical, behavioral, and cognitive parameters among control SAMR1 and SAMP8 and SAMP8 KL treated mice were depicted in a polygonal graph (Figure 2g).

**TABLE 1:** Parameters measured in the Open Field Test (OFT)

	SR1 Null	SP8 Null	SP8 s‐KL	SP8 m‐KL
Locomotor activity (cm)	1122.81 ± 43.01**	945.66 ± 35.65	1059.80 ± 39.91^#^	962.39 ± 47.53
Time in zone‐Center (s)	20.76 ± 2.38	15.49 ± 4.07	13.62 ± 4.17	21.36 ± 3.70
Time in zone‐Periphery (s)	279.24 ± 2.38	284.51 ± 4.07	286.38 ± 4.17	278.64 ± 3.70
Rearings (*n*)	21.78 ± 2.73	23.89 ± 3.11	29.36 ± 2.75	25.11 ± 3.55
Groomings (*n*)	2.33 ± 0.50*	1.00 ± 0.17	1.18 ± 0.18	1.44 ± 0.18
Defecations (*n*)	2.00 ± 0.58	1.89 ± 0.42	1.27 ± 0.38	1.11 ± 0.45
Urinations (*n*)	0.22 ± 0.15	0.33 ± 0.17	0.45 ± 0.16	0.11 ± 0.11

Note

*n*, a number of events. Results are expressed as a mean ± standard error of the mean (SEM).

* *p* < 0.05.

** *p* < 0.01 versus SR1 Null.

# *p* = 0.05 versus SP8 Null.

**TABLE 2:** Parameters measured in the Elevated Plus Maze Test (EPM)

	SR1 Null	SP8 Null	SP8 s‐KL	SP8 m‐KL
Total distance (cm)	1023.72 ± 75.82	1052.37 ± 64.41	1048.05 ± 60.01	1041.68 ± 49.37
Time in center zone (% test)	26.25 ± 3.40***	43.53 ± 2.45	38.49 ± 1.82	37.86 ± 1.56
Time in open arms (% test)	7.18 ± 2.70	16.40 ± 4.32	19.87 ± 3.15	19.47 ± 2.56
Time in closed arms (% test)	66.56 ± 5.64***	40.06 ± 2.65	41.64 ± 2.74	42.67 ± 3.02
Rearings (*n*)	15.33 ± 1.64	13.11 ± 0.98	13.00 ± 1.09	12.78 ± 1.05
Defecations (*n*)	1.78 ± 0.40***	0.00 ± 0.00	0.27 ± 0.19	0.00 ± 0.00
Urinations (*n*)	0.33 ± 0.17	0.00 ± 0.00	0.09 ± 0.09	0.11 ± 0.11

Note

*n*, a number of events. Results are expressed as a mean ± standard error of the mean (SEM).

*** *p* < 0.001 versus SR1 Null.

### 3.3 KL treatment rescues global epigenetic landscape in SAMP8 mice

**FIGURE 3:**
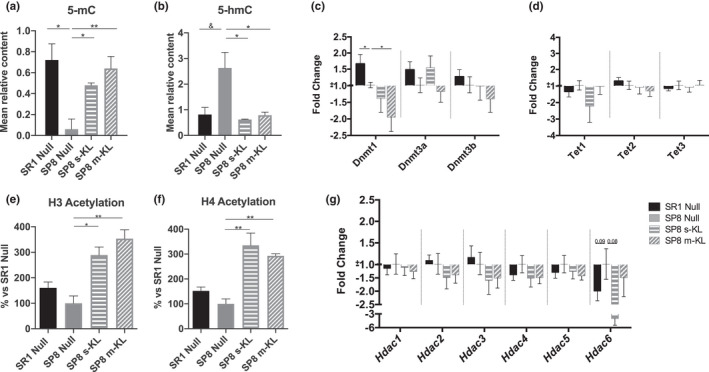
Global DNA methylation (a) and hydroxymethylation (b) levels measured using antibodies‐based colorimetric methods. The results are presented as the mean relative content of analyzed markers. Gene expression of enzymes involved in DNA methylation (c) and hydroxymethylation (d) expressed as fold change compared with SP8 Null. Global acetylation levels of histones H3 (e) and H4 (f), presented as % of SP8 Null acetylation levels. (g) Gene expression of enzymes involved in histone deacetylation expressed as fold change compared with SP8 Null. Mean ± standard error of the mean (SEM), *n* = 3–6. &*p* = 0.052; *p < 0.05 **p < 0.01

Dysregulation of global epigenetic markers provokes genomic instability and alterations in gene expression. This effect is increased during aging as well, as seen in models resembling the aging phenotypes. As expected, SAMP8 Null animals had global hypomethylation and increased hydroxymethylation. Remarkably, both s‐KL and m‐KL treatments rescued the global 5‐mC (Figure 3a) and 5‐hmC (Figure 3b) levels of SAMP8 mice to control SAMR1 values. In contrast, those changes modestly correlate with modification in the transcription of chromatin‐modifying enzymes involved in these epigenetic markers. This is the case of the DNA (cytosine‐5)‐methyltransferase family (DNMTs), where we only find a significantly reduced *Dnmt1* expression in m‐KL treated animals. (Figure 3c). Furthermore, no significant changes of gene expression were found in *ten‐eleven translocase family (TETs)* (Figure 3d). On the contrary, we found increased histone H3 (Figure 3e) and H4 (Figure 3f) acetylation levels in both s‐KL and m‐KL SAMP8 groups in comparison with decreased levels in SAMP8 Null group, without significant changes in *histone deacetylase enzymes (Hdac) 1*–*5*, despite a strong tendency that was observed for *Hdac6* (Figure 3g).

### 3.4 KL treatment affects histological and biochemical markers of SAMP8 animals

**FIGURE 4:**
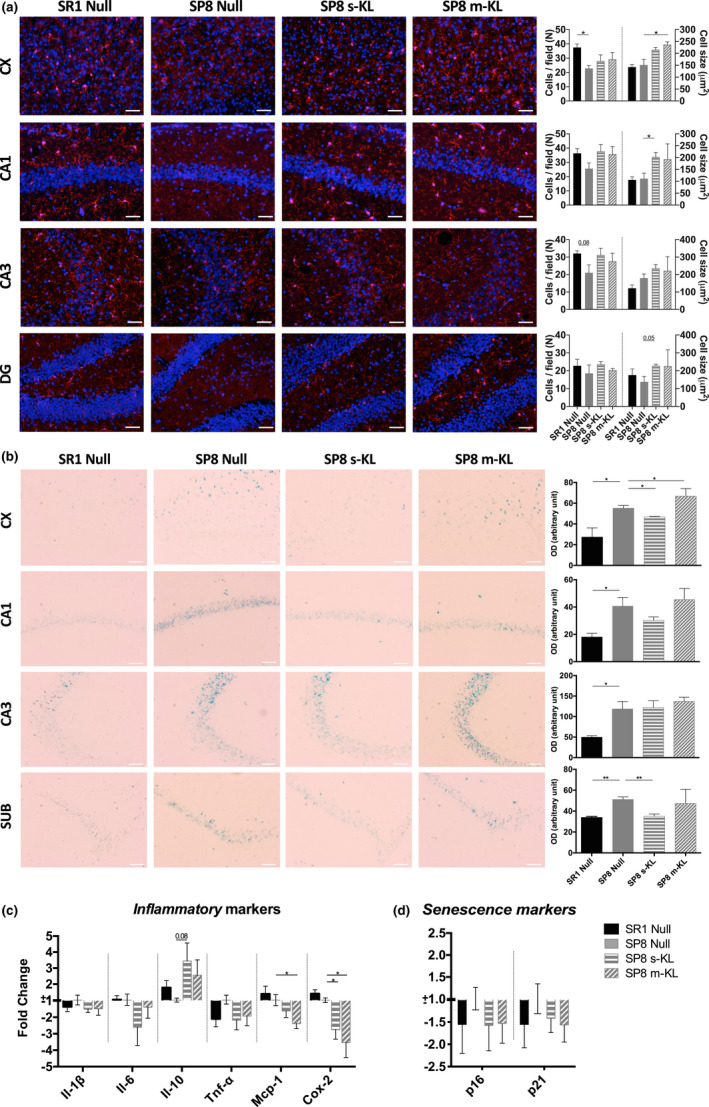
Immunofluorescence analysis of brain slices Iba1 of the Cortex (CX), CA1, CA3, and dentate gyrus (DG). (a) Scale bar for images 50 μm. Value measured as mean number of cells per field (first graph) and mean cell size in μm^2^ (second graph). Mean ± standard error of the mean (SEM), *n* = 3. (b) SA β‐gal activity staining of CX, CA1, CA2, and subiculum (SUB). Scale bar for images 50 μm. The value measured as optical density (OD) per image (arbitrary unit). Mean ± Standard error of the mean (SEM), *n* = 3. qPCR analysis of gene expression of inflammatory (c) and senescence markers (d) in the hippocampus area. Mean ± standard error of the mean (SEM), *n* = 5–6; *p < 0.05

Effects of KL treatment in the brain were studied both by histology and gene expression. Secreted and transmembrane isoforms presented similar effects in the histological variables studied. As shown in Figure 4a, the number of the inflammation marker Ionized calcium‐binding adaptor molecule 1 (Iba1) expressing cells was lower (or showed a tendency) in all the areas analyzed in SAMP8 mice compared with control SAMR1. Interestingly, both s‐KL and m‐KL treatments statistically increased the size of Iba1+ cells and tended to present similar cell number to SAMR1. A similar effect was observed for the astrocyte marker glial fibrillary acidic protein (GFAP) (Figure S1), where the number of cells in SAMR1 was similar to SAMP8 Null, but the average size was reduced between 20% and 25%. After treatment, the average size of GFAP+ cells reached SAMR1 mice values, being more evident in CA1 and DG than in CA3. In accordance with these results, gene expression of *Il*‐*1β* and *Tnf*‐*α* in SAMP8 Klotho‐treated animals mimicked the healthy control SAMR1 group (Figure 4c). Interestingly, after both KL treatments, expression of pro‐inflammatory proteins *monocyte chemoattractant protein*‐*1 (MCP*‐*1)* and *cyclooxygenase 2 (COX*‐*2)* was significantly decreased, while the expression of the anti‐inflammatory cytokine IL‐10 showed a clear tendency to increase.

Additionally, the activity of senescence‐associated β‐galactosidase (SA β‐gal) was assessed, and a reduction in global β‐gal enzyme activity was observed in subiculum and cortex after treatment with s‐KL (Figure 4b). Similar effects were observed for β‐galactosidase enzyme accumulation; both KL isoforms tended to recover enzyme levels in SP8 mice presenting levels equivalent to SAMR1 mice (Figure S2). Further senescence‐associated markers were analyzed, and KL treated groups tended to present lower p16 and p21 expression levels, similarly to SAMR1 mice (Figure 4d).

### 3.5 KL treatment rescues structural changes observed in aged SAMP8 tibia

**FIGURE 5:**
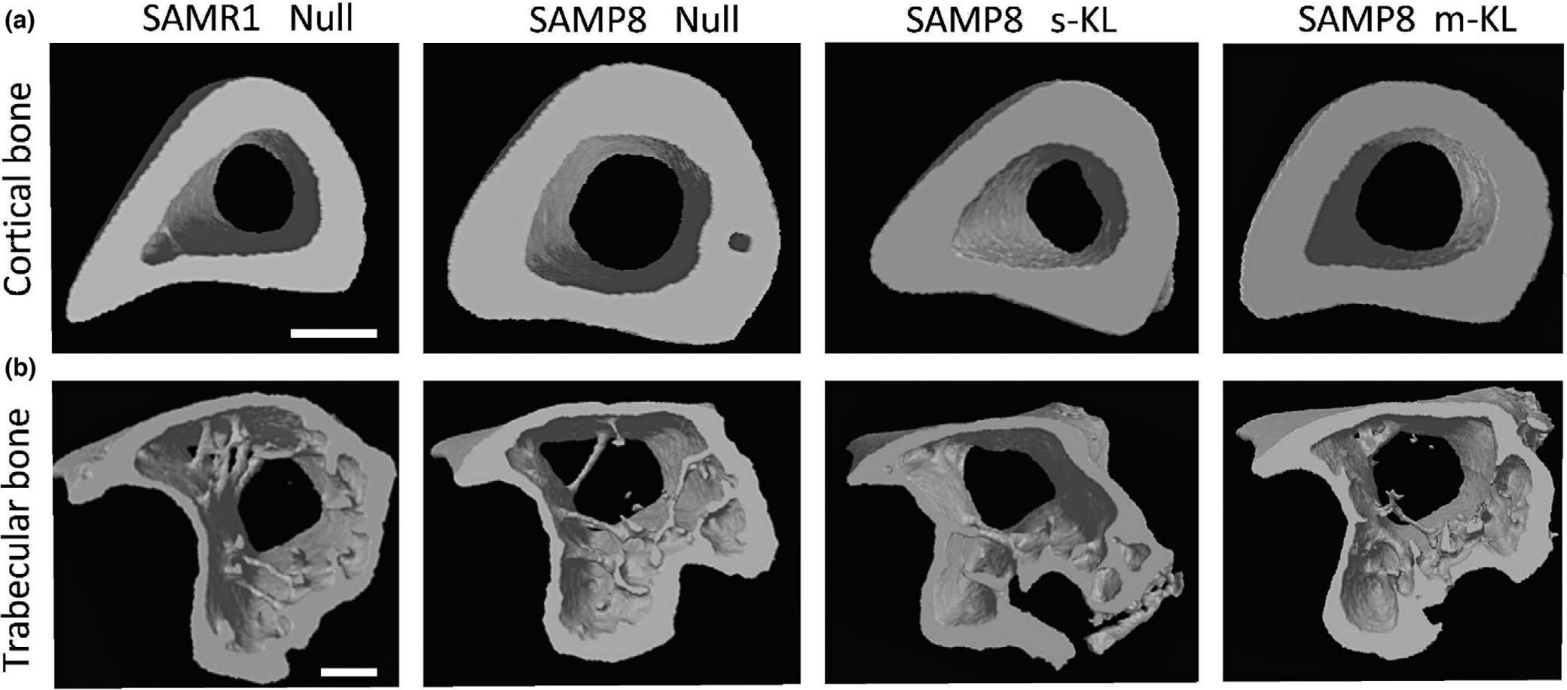
Bone microstructure analysis using MicroCT. (a) 3D Cortical bone representation of control and Klotho‐treated mice. (b) 3D Trabecular bone representation of tibial epiphysis. Scale bar = 0.5 mm

Effects of KL treatment over the microstructure of the long bone tibia (Figure 5) are quantified in Table 3. Mid‐tibial cortical bone presented a diameter expansion in SAMP8 Null mice, corresponding to a 5% perimeter and 16% cortical area expansion of SAMP8 compared with SAMR1 cortical area. Likewise, SAMP8 medullary space was also increased by 6% and 17% perimeter and endocortical area, respectively. Of note, Klotho treatment significantly prevented cortical expansion, showing parameter values equivalent to those of SAMR1 animals (Table 3). Interestingly, a higher reduction in periosteal and endocortical perimeter was detected with s‐KL compared with m‐KL treatment, although it was not statistically different from SAMR1 control group. In addition, other important parameters for determining bone strength and elasticity such as bone length, cortical thickness, or bone mineral density were not altered between SAMP8 and SAMR1 mice, or after the treatment. Percentage of trabecular bone volume (BV/TV) was reduced in SAMP8 model compared with SAMR1 (Table 3), which is compatible with the bone loss associated to the strain, a feature not affected by the KL treatment (Figure 5b).

**TABLE 3:** 2D Structural analysis of tibial cortical and trabecular bone using MicroCT

	SR1 Null	SP8 Null	SP8 s‐KL	SP8 m‐KL
Periosteal perimeter (mm)	6.34 ± 0.24^a^	6.65 ± 0.10	6.11 ± 0.40^b^	6.35 ± 0.20
Cortical area (mm^2^)	0.75 ± 0.05^a^	0.87 ± 0.08	0.75 ± 0.06^b^	0.76 ± 0.06^b^
Cortical thickness	0.24 ± 0.01	0.25 ± 0.01	0.25 ± 0.01	0.24 ± 0.02
Endocortical perimeter (mm)	2.15 ± 0.10^a^	2.28 ± 0.05	2.00 ± 0.16^b^	2.18 ± 0.11^c^
Medullary area (mm^2^)	0.30 ± 0.03^a^	0.35 ± 0.02	0.27 ± 0.04^b^	0.32 ± 0.03
Cortical BMD	1.22 ± 0.07	1.23 ± 0.07	1.21 ± 0.10	1.18 ± 0.05
Tibial length (mm)	16.14 ± 0.23	16.24 ± 0.13	16.17 ± 0.20	16.26 ± 0.08
BV/TV (%)	3.66 ± 0.80	2.49 ± 0.83	2.16 ± 0.91	2.36 ± 0.86
Trabecular thickness (mm)	0.029 ± 0.002	0.030 ± 0.003	0.027 ± 0.004	0.031 ± 0.004
Trabecular number (1/mm)	1.23 ± 0.18^a^	0.82 ± 0.24	0.78 ± 0.27	0.76 ± 0.22
Trabecular space (mm)	0.79 ± 0.13	1.30 ± 0.50	1.35 ± 0.39	1.39 ± 0.45
Trabecular BMD	0.69 ± 0.02	0.77 ± 0.07	0.70 ± 0.08	0.71 ± 0.07

Note

Data expressed as mean ± SD.

^a^ Statistical differences *p* < 0.05 between SAMR1 and SAMP8 Null.

^b^ Statistical differences *p* < 0.05 between SAMP8 Null and treated SAMP8.

^c^ Statistical differences *p* < 0.05 between SAMP8 s‐KL and SAMP8 m‐KL.

The effect of secreted KL treatment was only detectable in SAMP8 mice, as SAMR1 animals treated with s‐KL did not show alterations in the analyzed cortical or trabecular parameters compared with control SAMR1 animals (Table S1). Relevance in bone strength of the detected structural alteration was assessed with a 3‐points bending fracture test (Figure S3). No differences were observed comparing SAMP8 treatment groups in the strength needed for bone fracture, although a tendency was observed to increased bone plasticity under stress with KL treatments, mainly with the m‐KL isoform.

## 4 DISCUSSION

The anti‐aging and cognition‐enhancing gene KL can act through its pleiotropic protective functions to counteract the diverse signaling cascades and mechanisms underlying senescence (Moos et al., 2020). Therapeutic approaches increasing *KL* gene expression can prevent further neurodegeneration and memory loss associated with aging and Alzheimer's disease (AD) (Abraham et al., 2016; Dubal et al., 2015). Although KL is mainly expressed in the kidney and does not cross the blood‐brain barrier (BBB) (Leon et al., 2017), it is also expressed in the brain (Masso et al., 2015; Moos et al., 2020). Our study used intracerebroventricular administration of AAVs as a delivery approach to increase KL expression in the CNS as a promising therapeutic strategy to normalize some relevant aging hallmarks present in SAMP8 mice. This procedure also allowed liver transduction due to viral vector extravasation from cerebroventricular fluid to the bloodstream after the injection (Schuster et al., 2014).

Our results revealed the beneficial effects of sustained KL expression by improving the physical condition and preserving memory in both working memory evaluated by NORT, and spatial memory tested by OLT. However, we did not find modifications in behavior and emotional assays in SAMP8 mice after KL treatment. KL molecular mechanisms are not entirely understood, but it has been described that KL increases synaptic plasticity and cognitive performance, modifying several structures, and brain functions during aging (Abraham et al., 2016).

In accordance with these results, a study developed by Zhou and collaborators demonstrated neuronal and cognitive beneficial effects of lentiviral‐mediated m‐KL expression on SAMP8 mice (Zhou et al., 2018). Furthermore, our group previously described that a single injection into the CNS of AAV9 expressing s‐KL improved learning and memory performance in 12‐month‐old C57BL/6J males, suggesting that s‐KL isoform activity and not just m‐KL, might be particularly important in the CNS (Masso et al., 2018). After comparing both isoforms, we obtained similar effects over memory and physical state behavioral tests. However, the biosafety profile of the s‐KL protein is better than that of m‐KL, due to the lack of KL2 domain, which is involved in bone mineral homeostasis, being this is a relevant consideration in the field of Klotho‐based therapies for aging associated diseases.

During aging, general epigenetic alterations are observed, resulting in a progressive global hypomethylation (in non‐CpG islands) together with increased local hypermethylation (primarily CpG islands) (Fraga et al., 2007; Lopez‐Otin et al., 2013), while hydroxymethylation is increased in non‐pathological aging, as well as in brains of aged transgenic AD mice (Cadena‐del‐Castilloa et al., 2014). Accordingly, in this study, that trend was replicated in the SAMP8 strain, previously associated with accelerated epigenetic changes compared with SAMR1 controls (Grinan‐Ferre et al., 2018). Strikingly, this typical SAMP8 phenotype was rescued after KL treatments, paralleling the 5‐mC levels of control SAMR1 mice. Although *Dnmt1* gene expression was reduced, expression of *Dnmt3a*/*b* genes was not modified. This is, however, in line with previous results in SAMP8 mice after environmental enrichment intervention, where increased methylation levels were associated with no change or even a reduction in the levels of different members of the DNMT and TET families (Grinan‐Ferre et al., 2016). Similarly, continuous s‐KL and m‐KL expression reduced age‐associated 5‐hmC levels, albeit without promoting any change in enzymes’ gene expression from the TETs family. These data are consistent with results reported recently by Irier and co‐workers in mice, where no modifications in TETs family enzymes gene expression were found after an intervention that promoted cognitive improvement (Irier et al., 2014).

Although the modulation of histone acetylases and HDACs in AD remains controversial, histone acetylation is another crucial epigenetic modification in the AD pathology. It has been reported that HDAC inhibition increased histone acetylation H3/H4 and rescued learning and memory deficits in AD animal models (Grinan‐Ferre et al., 2016; Lu et al., 2015). Here, we observed a significant increase in H3 and H4 acetylation levels after s‐KL or m‐KL single treatment in SAMP8 mice, whereas an evident, though no significant, reduction in HDACs family gene expression was found. In agreement with our results, the SAMP8 strain showed reduced H3/H4 acetylation levels when it was compared with age‐matched SAMR1, and the acetylation levels were higher after interventions that improved the cognitive performance of the SAMP8 mouse (Grinan‐Ferre et al., 2016).

Previous studies linked genetic KL variants, which produce lower circulating KL levels, with advanced epigenetic age, suggesting a direct or indirect role of KL over age‐related epigenetic status (Wolf et al., 2020). However, although many studies have associated the beneficial effects of KL in animal models (Dubal et al., 2015; Masso et al., 2018; Zhou et al., 2018), this is, to the best of our knowledge, the first time that KL has been linked to an intervention affecting global epigenetic mechanisms with resulting neuroprotective effects, including better physical condition and cognitive performance. In this context, we hypothesize that Klotho´s pleiotropic activities can finally lead to changes in the epigenetic landscape through different mechanisms either directly, by increasing epigenetic markers as 5‐mC levels; or indirectly, by altering gene expression of several chromatin‐modifying enzymes, such as *Dnmt1* and *Hdac6*.

It is well established that persistent inflammation plays a prominent role in aged and AD brain, exacerbating Aβ pathology, and tau hyperphosphorylation (Kinney et al., 2018). Previous studies have also shown that KL suppresses several inflammatory markers such as TNF‐α (Maekawa et al., 2009), while KL depletion contributes to increased inflammation in a mouse model (Zhao et al., 2011), confirming the modulation induced by KL and suggesting that an interaction occurs between KL and inflammation. Here, we observed a tendency in increased gene expression of the anti‐inflammatory cytokine *Il*‐*10* and a significant reduction in the expression of several pro‐inflammatory markers such as *Mcp*‐*1*, *Cox*‐*2*, and a clear tendency to a reduced *Tnf*‐*α* and *Il*‐*6* in s‐KL or m‐KL treated SAMP8 mice. Moreover, a recovery in levels of inflammation‐related markers was observed after an immunofluorescence study of different brain areas in treated mice. Previous studies in aged mice reported decreased Iba1+ microglial cell number and arborization, especially in the cortex (Zoller et al., 2018). Similarly, progressive Iba1+ cell arborization loss was observed in human post‐mortem samples during aging and AD progression (Davies et al., 2017). In this study, both s‐KL and m‐KL treatments statistically increased the size of Iba1+ cells while the average size of GFAP+ cells resembled the size in SAMR1 mice. Regarding the cell morphology, the changes were much more evident in GFAP+ cells, were s‐KL and m‐KL treatments were associated with greater cell processes thickness including those cells surrounding the vessels. In addition, SA β‐Gal has been widely used to address neuronal wellness during aging since an accumulation and increased activity of this enzyme have been described in senescent cells (Geng et al., 2010). Strikingly, after the two months treatment, both isoforms of KL reduced the accumulation of the enzyme, although only the s‐KL isoform reduced SA β‐Gal activity in a statistically significant manner. More studies are needed to depict if this effect is due to β‐Gal breakdown or inhibition of the protein accumulation due to reduced cellular stress.

Previous bone studies have reported age‐related geometric remodeling, tending to a medullary space and cortical perimeter expansion, due to endocortical bone absorption and periosteal bone apposition (Chen et al., 2009; Riggs et al., 2004). This cortical bone expansion has been described in long bones of human and animal models for osteoporosis research, and it is explained as a compensatory mechanism to increase bone strength under bone loss, alterations in trabecular connectivity, and low protein intake circumstances (Ammann & Rizzoli, 2003). Similarly, cortical expansion was detected in male C57BL/6 and senescence‐accelerated‐mouse prone strains (SAMP), although it did not counteract increased bone weakness in aged individuals (Ferguson et al., 2003; Silva et al., 2002). In our study, this tendency is reproduced in SAMP8 Null animals, which exhibit a tibial cortical bone expansion. Interestingly, this effect is abolished after two months expression of the KL isoforms. The cortical expansion was only observed in SAMP8 bones, as SAMR1 animals treated with AAV9 s‐KL did not exhibit altered bone parameters. Silva et al. reported that SAMP6 tibial cortical and medullar cavity expansion correlated with decreased resistance to fracture, with no BMD parameter changes (Silva et al., 2002). That correlation was not reproduced in our study using SAMP8 mice as no differences in breaking force were observed after the treatment. Interestingly, Klotho‐treated animals presented a tendency to increase bone displacement prior to fracture. In this regard, previous studies have reported a positive effect of Klotho in maintaining bone structure (Maruyama et al., 2015) and composition protection under normal and disease conditions (Lin et al., 2017), directly through FGF23 signaling and, indirectly, by modulating the function of ion transporters and reducing cellular damage. In contrast, low KL levels generate an osteoporotic phenotype characterized by blood ion deregulation, low bone turnover, and reduced fracture resistance (Maruyama et al., 2015; Sasaki et al., 2013). More specific analyses are needed to fully understand the molecular mechanisms used by KL isoforms to affect bone structure and the other parameters analyzed.

From a comparative point of view, both KL isoforms resulted in efficient improvement of cognitive and physical deficits associated with aged SAMP8 mice. Interestingly, just s‐KL isoform resulted in body weight maintenance during the experiment and increased horizontal activity on the OF test. Both isoforms efficiently rescued global epigenetic levels associated with younger animals, a property not previously described before for this protein. The anti‐inflammatory properties were also comparable, although just s‐KL efficiently reduced SA β‐Gal activity in CX and SUB areas. These observed differences could be related to an increased availability or diffusion of s‐KL isoform due to its putative secreted form and reduced molecular weight compared to m‐KL isoform. Finally, the effect over bone structure was also comparable after treatment with both isoforms.

In summary, our in vivo results after a single treatment of AAVs expressing s‐KL in SAMP8 mice revealed new potential therapeutic properties of KL by improving age‐related deficits and relevant molecular aging hallmarks such as epigenetic, inflammatory, and bone alterations. Moreover, it reemphasized the therapeutic potential of the s‐KL isoform in CNS and physical status, as well as its isoform‐specific effects, probably due to increased protein diffusion.

## CONFLICT OF INTEREST

Portions of this work are the subject of a patent application held by the Universitat Autonoma de Barcelona (UAB, Spain); the Universitat de Barcelona (UB, Spain); the Institucio Catalana de Recerca i Estudis Avançats (ICREA, Spain); and the Vall d'Hebron Institute of Research (VHIR, Spain).

## AUTHOR CONTRIBUTIONS

J. Roig‐Soriano, C. Griñán‐Ferré, M. Pallàs, J. F. Espinosa‐Parrilla, A. Bosch, and Miguel Chillon designed research. J. Roig‐Soriano, and C. Griñán‐Ferré performed research. J. Roig‐Soriano, C. Griñán‐Ferré, J. F. Espinosa‐Parrilla, A. Bosch, C. R. Abraham, and Miguel Chillon analyzed and interpreted the data. J. Roig‐Soriano and C. Griñán‐Ferré wrote the paper. Miguel Chillon is the corresponding author. All authors read and approved the final manuscript.

## Supporting information

Fig S1

Fig S2

Fig S3

Table S1‐S3

## Data Availability

The data that support the findings of this study are available from the corresponding author upon reasonable request.
