# Supplementary figures and images for: AAV‐mediated expression of secreted and transmembrane αKlotho isoforms rescues relevant aging hallmarks in senescent SAMP8 mice
Source: Aging Cell. 2022 Mar 10;21(4):e13581. doi: 10.1111/acel.13581 (PMC9009104; doi:10.1111/acel.13581)

CA1

SR1 Null

SP8 Null

SP8 s-KL

SP8 m-KL

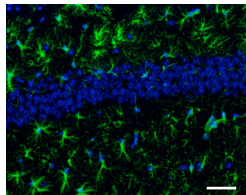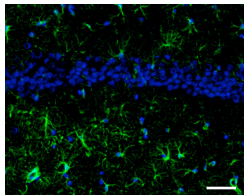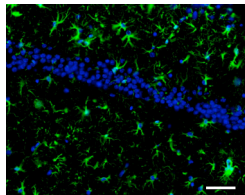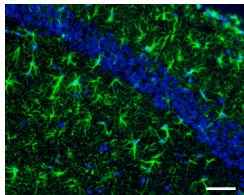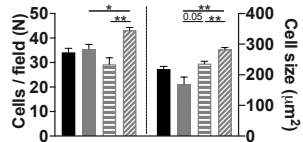

CA3

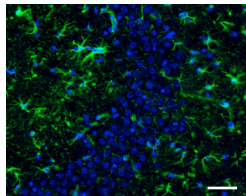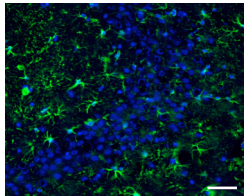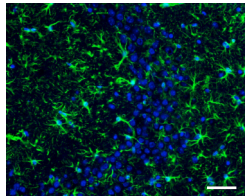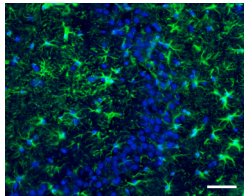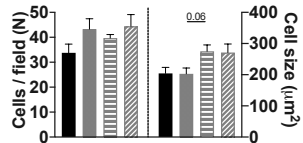

DG

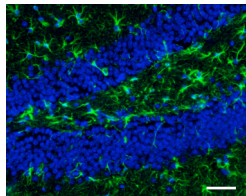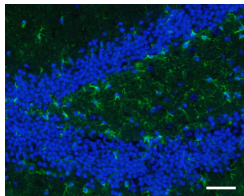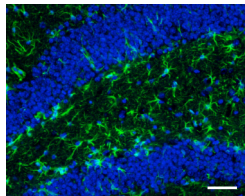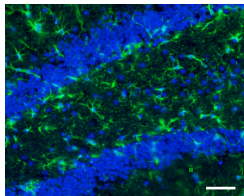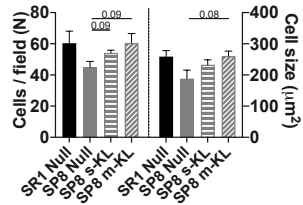

Supplement: Supplementary file 1 — Fig S1 [file ACEL-21-e13581-s002.pdf]

SR1 Null

SP8 Null

SP8 s-KL

SP8 m-KL

CX

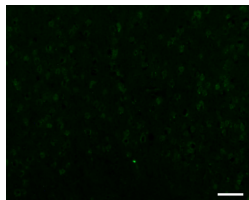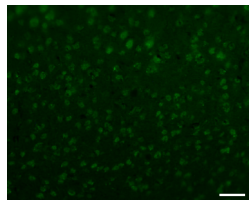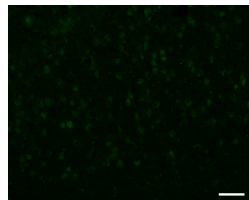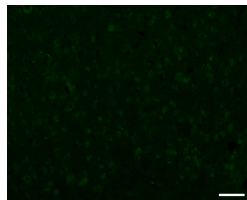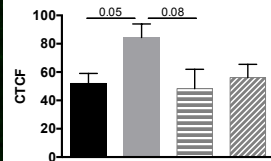

CA1

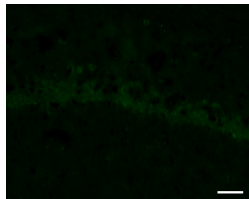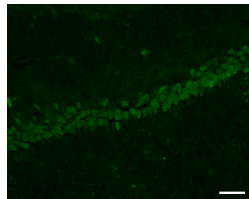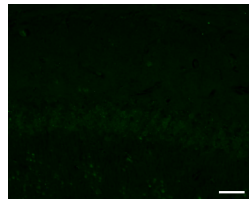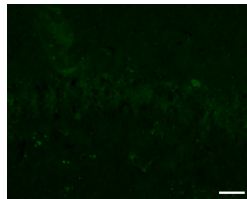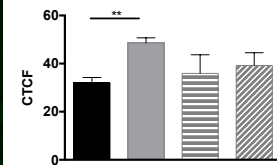

CA3

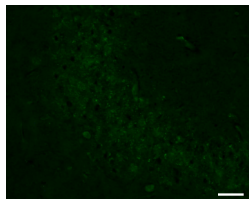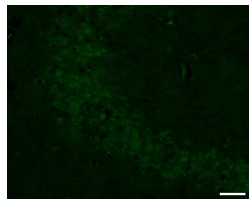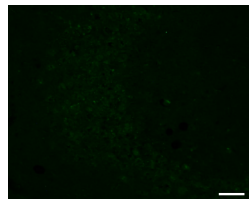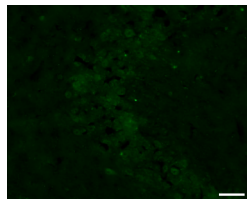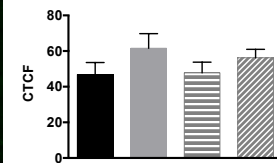

DG

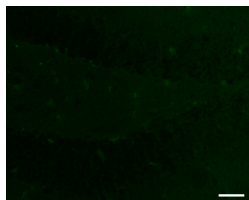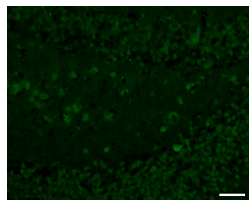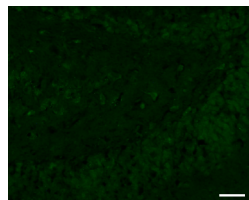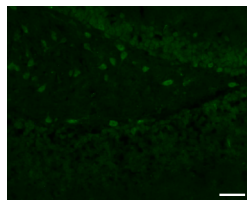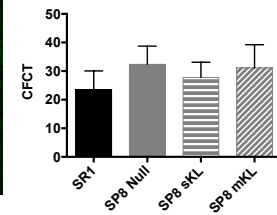

Supplement: Supplementary file 2 — Fig S2 [file ACEL-21-e13581-s004.pdf]

**a**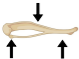**b**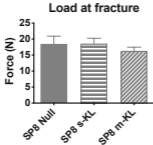**c**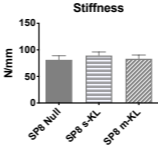**d**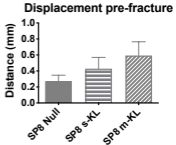

Supplement: Supplementary file 3 — Fig S3 [file ACEL-21-e13581-s001.pdf]
