# Supplementary material for: AAV‐mediated expression of secreted and transmembrane αKlotho isoforms rescues relevant aging hallmarks in senescent SAMP8 mice
Source: Aging Cell. 2022 Mar 10;21(4):e13581. doi: 10.1111/acel.13581 (PMC9009104; doi:10.1111/acel.13581)
Supplement: Supplementary file 4 — Table S1‐S3 [file ACEL-21-e13581-s003.docx]

Supplementary Table 1. 2D Structural analysis of tibial cortical and trabecular bone using MicroCT. Data expressed as mean ± SD.

|  | SR1 Null | SR1 s-KL |
| --- | --- | --- |
| Periosteal perimeter (mm) | 6.34 ± 0.24 | 6.24 ± 0.30 |
| Cortical area (mm^2^) | 0.75 ± 0.05 | 0.74 ± 0.06 |
| Cortical thickness | 0.24 ± 0.01 | 0.24 ± 0.01 |
| Endocortical perimeter (mm) | 2.15 ± 0.10 | 2.12 ± 0.16 |
| Medullary area (mm^2^) | 0.30 ± 0.03 | 0.30 ± 0.05 |
| Cortical BMD | 1.22 ± 0.07 | 1.22 ± 0.06 |
| Tibial length (mm) | 16.14 ± 0.24 | 16.36 ± 0.10 |
| BV/TV (%) | 3.66 ± 0.80 | 3.31 ± 1.82 |
| Trabecular thickness (mm) | 0.029 ± 0.002 | 0.030 ± 0.002 |
| Trabecular number (1/mm) | 1.23 ± 0.18 | 1.07 ± 0.55 |
| Trabecular space (mm) | 0.79 ± 0.13 | 1.18 ± 0.59 |
| Trabecular BMD | 0.69 ± 0.02 | 0.70 ± 0.02 |

Supplementary Table 2. Primers used in qPCR studies. SYBR Green primers.

| Target | Product size (bp) | Forward primer (5’-3’) | Reverse primer (5’-3’) |
| --- | --- | --- | --- |
| *Cox2* | 126 | TGACCCCCAAGGCTCAAATA | CCCAGGTCCTCGCTTATGATC |
| *Dnmt1* | 85 | ACCTGGAGAGCAGAAATGGC | TGAAAGGGTGTCACTGTCCG |
| *Dnmt3a* | 269 | GAGCCGCCTGAAGCCC | TCTTCCTTGCCACGGTTCTC |
| *Dnmt3b* | 142 | TGCCAGACCTTGGAAACCTC | GCTGGCACCCTCTTCTTCAT |
| *Hdac1* | 150 | TCACCGAATCCGCATGACTC | TCTGGGCGAATAGAACGCAG |
| *Hdac2* | 280 | CTATCCCGCTCTGTGCCCT | GAGGCTTCATGGGATGACCC |
| *Hdac3* | 97 | GCACCCAGTGTCCAGATTCA | AGTTCTCCTCGGGACCTCTC |
| *Hdac4* | 290 | GACTCGAGAAGGCTCAGTCG | CTGTGACAAGGGGTGTCTGG |
| *Hdac5* | 296 | CTGGACAGTTCCCCAAACCA | GTCCAGGAGCAAACGTGC |
| *Hdac6* | 294 | AGCCTGGTTAAACGGTAGGC | AAGGCTCTCTAATCTGCGCC |
| *Il-1β* | 101 | ACAGAATATCAACCAACAAGTGATATTCTC | GATTCTTTCCTTTGAGGCCCA |
| *Il-6* | 189 | ATCCAGTTGCCTTCTTGGGACTGA | TAAGCCTCCGACTTGTGAAGTGGT |
| *Il-10* | 175 | GGCGCTGTCATCGATTTCTCCCC | TGGCCTTGTAGACACCTTGGTCTT |
| *Mcp1* | 197 | CCCACTCACCTGCTGCTACT | TCTGGACCCATTCCTTCTTG |
| *p16* | 364 | AACTCTTTCGGTCGTACCCC | GCGTGCTTGAGCTGAAGCTA |
| *p21* | 122 | TTGTCGCTGTCTTGCACTCTGGT | CTGCGCTTGGAGTGAT |
| *Tet1* | 188 | CTGCCAACTACCCCAAACTCA | TCGGGGTTTTGTCTTCCGTT |
| *Tet2* | 113 | CCATCATGTTGTGGGACGGA | ATTCTGAGAACAGCGACGGT |
| *Tet3* | 109 | GGGCAGGCAGCGTAGC | ATGAGGTGAGCCAATGGGTG |
| *Tnf-α* | 139 | TCGGGGTGATCGGTCCCCAA | TGGTTTGCTACGACGTGGGCT |
| *s-KL* | 315 | TCATAATGGAAACCTTAAAAGCAA | CACTGGGTTTTGTCAAAGGA |
| *m-KL* | 157 | TACGGAGACCTCCCGATGTA | CGCAAAGTAGCCACAAAGGT |
| *β-actin* | 190 | CAACGAGCGGTTCCGAT | GCCACAGGTTCCATACCCA |

Supplementary Table 3. Antibodies used in Immunofluorescence studies.

| Antibody | Host | Source/Catalog | Dilution |
| --- | --- | --- | --- |
| β-galactosidase | Rabbit | Abcam/AB616 | 1:1000 |
| IBA1 | Goat | Abcam/ab5076 | 1:200 |
| GFAP | Rabbit | Dako/GA524 | 1:200 |
| αKlotho | Goat | Abcam/BAF1819 | 1:100 |
| Alexa Fluor® 568 anti-goat | Donkey | Invitrogen/A21206 | 1:500 |
| Alexa Fluor® 488 anti-rabbit | Donkey | Invitrogen/A11057 | 1:800 |
| Alexa Fluor® 568 Streptavidin |  | Invitrogen/S11226 | 1:200 |
